# Danish guidelines for treating acute colonic obstruction caused by colorectal cancer—a review

**DOI:** 10.3389/fsurg.2024.1400814

**Published:** 2024-11-19

**Authors:** Martynas Mikalonis, Tue Højslev Avlund, Uffe Schou Løve

**Affiliations:** ^1^ Department of Surgery, Regional Hospital Viborg, Viborg, Denmark; ^2^ Department of Surgery, Aarhus University Hospital, Aarhus, Denmark

**Keywords:** colorectal cancer, colonic obstruction, emergency, guidelines, surgery

## Abstract

Acute onset of colonic obstruction caused by colorectal cancer occurs in approximately 14% of Danish patients with colon cancer(1). Given that colorectal cancer is a common cancer with about 4,500 new cases annually, acute onset will occur in a reasonably large number of patients in Danish emergency departments, and all surgeons should be familiar with the treatment principles. A revised guideline from the Danish Colorectal Cancer Group is currently underway, and this status article reviews the latest knowledge and recommendations.

## Introduction

Bowel obstruction as a first symptom is observed in approximately 14% of Danish patients with colon cancer according to previous Danish Colorectal Cancer Group (DCCG) annual reports (1). Understanding the treatment options for acute colonic cancer obstruction is crucial for timely intervention and improved patient outcomes.

Traditionally, emergency surgery has been performed to treat patients with acute colonic obstruction. Emergency surgery for colon cancer is still associated with high 30- and 90-day mortality rates. Changing from emergency to elective surgery for the treatment of acute colonic obstruction without perforation seems to be desirable. This approach will enable preoperative staging, optimization and planning of the procedure. Patients with metastatic cancer without signs of bowel perforation can be spared surgery.

Another feared complication associated with high morbidity and mortality rates is colonic perforation. The risk of perforation of the coecum in patients with colonic ileus increases with radiological findings of a coecum diameter ≥12 cm, and urgent decompression is recommended (2).

These guidelines address the management of large bowel obstruction in patients with colorectal cancer. The overall purpose of these guidelines is to provide uniform, high quality evidence-based cancer treatment across Denmark. These guidelines are primarily intended to support clinical work and the development of clinical treatment quality, which is why the primary target group in the Danish health care system is health care professionals (surgeons, oncologists, primary health care physicians, policy-makers).

Recently, updated ASCRS guidelines provide similar management recommendations for patients with right-sided and left-sided colonic obstruction as well as for those with colonic perforation (3).

## Materials and methods

A literature review was performed using PubMed articles from 2010–2022 with the search string “Intestinal Obstruction” [Mesh]) OR (“bowel obstruction” OR “obstruction” OR “colon obstruction” OR “intestinal obstruction”). Available literature from the PubMed, Cochrane and Embase electronic databases was used for the section on the treatment of perforation. The search strategy was as follows: (Colon cancer OR Rectal cancer OR colorectal cancer) AND Perforation AND surgery AND acute AND emergency. Only articles in English were searched for. Any potential conflicts were resolved through discussion after the screening results were revealed, and if any disagreements persisted, the systematic review coordinator made the final decision. The same reviewers conducted a full-text screening of the selected articles. The Oxford 2009 Levels of Evidence were used to determine levels of evidence and levels of recommendation.

## Results and discussion

### Diagnostics

In acute colonic obstruction, a computed tomography (CT) scan of the abdomen with IV contrast should be performed (B).

The diagnosis of colonic ileus can be made by CT scan, which has a high sensitivity (91%) and specificity (91%) (4) [2B]. CT scans can be used to identify the anatomical localization of the obstruction and assess the severity of the ileus based on the diameter of the cecum. In addition, CT scans with intravenous contrast can often clarify the cause of ileus, identify signs of ischemia and help surgeons determine the stage of a potential tumor. This allows the treatment strategy and its timing to be planned more effectively (5) [4]. CT scans can be supplemented with contrast enema to further clarify the completeness of the stenosis. Therefore, CT scan appears to be superior to conventional x-ray imaging (6) [2B].

### Acute surgical treatment

The treatment strategy for colonic obstruction should be determined by a colorectal surgeon. Surgical resection should be performed with the participation of a colorectal surgeon (B).

If possible, treatment of colonic obstruction should be performed during the day and with the participation of a colorectal surgeon. The morbidity, mortality and anastomotic leakage rates are likely to be lower when surgery is performed by an experienced colorectal surgeon (7–11) [2A–B]. Long-term survival after emergency colorectal resection for cancer is also likely related to surgeon subspecialization (7, 8, 12) [2A–B]. A Swedish registry study failed to demonstrate differences in survival between patients treated by emergency surgeons and those treated by colorectal surgeons but the registry study still showed an increased rate of permanent stomas in patients operated on by emergency surgeons (13) [2B].

The following treatment modalities for colonic obstruction are equivalent in terms of survival: stenting, colonic stoma placement and resection with or without primary anastomosis (A).

The treatment strategy for obstructing colorectal cancer depends on the patient's clinical condition and tumor location. Emergency surgery for colon cancer is still associated with high 30- and 90-day mortality rates. In a Swedish report from 2014, the 30- and 90-day mortality rates were 8.2 and 14.9%, respectively (14) [2B], while the Danish 30-day mortality, in emergency setting, in a DCCG theme report from 2018 was 12%.

In the case of acute resection, both morbidity and mortality are higher than those of elective resection (14) [2B], and there is a greater risk of colostomy (15) [1B]. Changing from emergency to elective surgery for the treatment of acute colonic obstruction without perforation seems to be desirable. This approach will enable preoperative staging, optimization and planning of the procedure. Patients with metastatic cancer without signs of bowel perforation can be spared surgery.

#### Colonic stenting for left-sided malignant colonic obstruction

There are a large number of publications on short-term outcomes after decompression via self-expandable metallic stenting in the colon accounting for the feasibility of procedure. According to a 2017 meta-analysis of 448 patients from seven randomized trials comparing stenting as a bridge to surgery and emergency colon resection for left-sided colorectal cancer, the stent group had lower rates of permanent stoma and lower morbidity. Patients receiving primary anastomosis in the stent group accounted for 71.7% vs. 55.3% in acute resection group (RR 1.27 95pct. CI (0.98–1.64). There was no difference in mortality or anastomotic leakage rate (16) [1A]. Similar results have been reported from other meta-analyses of randomized trials (17–20) [1A].

A retrospective study comparing results after self-expanding metallic stents vs. stoma decompression exhibited financial savings and shorter hospitalization times in the stent group but no difference in the clinical success rate in terms of obstruction resolution (21) [3B]. A recent randomized English study of colon cancer patients presenting with colonic obstruction requiring stenting showed no difference in morbidity, mortality, or 3-year disease-free survival (DFS) between patients treated with stents and patients treated with acute resection or stoma placement (22) [1B]. However, another recent meta-analysis of randomized trials showed significantly lower permanent stoma rates in the stenting group than in the acute resection group. Moreover, significantly lower morbidity but not significantly lower mortality was shown (23) [1A].

According to a Cochrane meta-analysis, stent-related complications were described as acceptable (stent-related perforation 5.8%, stent migration 2.1% and stent obstruction 2.1%) (24) [1A]. A more recent Danish study reported a stent perforation rate of 8.9 (25) [2B]. In a systematic review of 82 studies (2,287 patients), Datye et al. (26) [3A] failed to observe a significant difference in perforation rates between patients who underwent stenting in a palliative setting and patients who underwent bridging to surgery. The overall perforation rate was 4.9%, approximately half of which occurred in the first 24 h. The risk factors for perforation were chemotherapy, radiotherapy and glucocorticoid therapy. The mortality rate among patients with perforation was 16.2%. The degree of obstruction should be taken into account when evaluating perforation risk. A retrospective review of 130 patients reported that the perforation rate is associated with the angle of stenosis (27) [2B], a factor that should also be considered before stent placement. Three randomized trials from 2008–2011 described asymptomatic perforation rates ranging from 6%–27% (28–30) [1A], which has raised concerns about the long-term outcomes of the placement of a metallic stent as a bridge to surgery. In addition to the risk of perforation, colon cancer stenting may theoretically have other oncological disadvantages due to pressure on the tumor. A 2021 meta-analysis by Balciscueta et al. found an increased incidence of perineural ingrowth and lymphatic vessel ingrowth in patients who underwent stenting as a bridge to surgery compared to that in patients who underwent urgent resection (31) [2A]. The same author's 2020 meta-analysis found increased local recurrence rates in patients with stent-related perforation but no difference in 3- or 5-year survival (32) [2A]. On the other hand, another retrospective study from Italy showed no difference in perineural ingrowth between stented vs. primarily resected tumors (33) [2B]. Other recent studies also failed to demonstrate lower long-term survival with stenting than with emergency surgery. Two Spanish studies showed no difference in 3-year DFS (34, 35) [2B]. Thus, the data are inconclusive, and no conclusion can currently be drawn on long-term survival.

#### Colonic stenting for right-sided malignant colonic obstruction

Recent retrospective studies have shown similar morbidity and mortality for right-sided stenting vs. emergency surgery, as well as a lower rate of stoma formation (36) [3B]. A systematic review of 14 cohort studies from 2015 reported less overall morbidity and mortality for stenting than for emergency resection of acute right-sided colonic obstruction and a lower rate of stoma formation (37) [2A]. This was confirmed in new meta-analyses from 2022 (38) [2A] and 2021 (39) [2A]. Stenting of colonic tumors proximal to the splenic flexure can thus be performed at centers where expertise is available.

The optimal timing of surgery after stenting has not been well described, but evidence suggests that surgery should be performed as soon as possible after the patient's condition has stabilized and the necessary assessment has been performed. The ESGE guidelines recommend surgery approximately 14 days after stent placement (40). This finding is supported by a Danish study that showed that increased recurrence rates were associated with long intervals between stent placement and surgery (41) [2B].

#### Decompressing stoma

A meta-analysis of 8 studies comparing temporary stoma placement vs. emergency surgical resection found no difference in 30-day morbidity or mortality, which was approximately 7% for both groups. There were fewer permanent stomas in the decompressing stoma group (42) [2A]. Due to concerns about long-term outcomes after stenting as a bridge to surgery, there has been a focus on this topic in recent years. A 2016 cohort study comparing stents vs. stomas as a bridge to resection found fewer required procedures and lower long-term morbidity (primarily due to herniation) in the stent group (43) [2B] but otherwise comparable outcomes. A more recent meta-analysis from 2022 comparing stents vs. stomas showed no difference in 3-year overall survival (OS), perioperative mortality or permanent stoma rates. However, there were fewer Clavien‒Dindo 1–2 complications in the stent group but similar Clavien‒Dindo 3–4 complications in both groups, and there was no difference in the permanent stoma rate (44). Another meta-analysis from 2021 comprising 48 studies, including 8 randomized studies, examined 5-year OS with stenting or stoma placement as a bridge to surgery. A significantly higher 5-year OS was associated with stoma placement than with stenting, but conversely, stoma placement was associated with a longer hospitalization time (45). Therefore, decompressing stoma placement cannot be dismissed as a good alternative to stenting as a bridge to surgery. This should especially be considered in patients with long remaining life expectancies.

#### Emergency resection

For right-sided colonic obstruction (acute obstructing tumors orally to the splenic flexure) without feculent peritonitis, right-sided hemicolectomy with primary ileocolic anastomosis can be safely performed in selected patients. In Denmark, the leakage rate after acute colonic resection is 2.8%, according to the DCCG annual report from 2012 (1) [2C]. The anastomotic leakage rate has been reported to be between 2.5 and 5.2% in retrospective studies of acute right-sided colon resection (46, 47) [2B]. For left-sided colonic obstruction (acute obstructing tumors in or anally to the splenic flexure), primary resection can be performed with an anastomosis with manual emptying of the dilated colon orally to the tumor. In a systematic review, Kam et al. reported a significantly higher anastomotic leakage rate (7%) in patients who underwent antegrade lavage than in those who underwent manual emptying (1%). There was a significantly higher 30-day mortality after antegrade irrigation (7.2% vs. 1%) (48) [2A]. Hartmann's operation is a preferred surgical strategy for patients at high risk of anastomotic leakage. Colectomy can be performed for severely distended and damaged colons or in the presence of synchronous colon tumors (49) [2A].

In palliative treatment of colonic obstruction, stenting is the first choice where technically feasible (B).

With stent placement, patients with metastatic disease avoid a stoma and the following reduced quality of life. Meisner et al. (50) [2B] demonstrated a 98.4% technical success rate, 87.8% clinical success rate and low complication rate (perforation 5.1%, migration 5.5%) in a prospective multicenter study of stenting in a palliative setting. The mortality rate was less than 2% (two patients died—one after 24 days and one after 34 days). The risk of perforation seems to be an unresolved issue. A Dutch randomized trial comparing acute resection with stenting for palliation of mechanical ileus in patients with metastatic colon cancer was stopped early due to a high rate of stent-related perforations (6 perforations out of 11 stents placed) (28) [1B]. This perforation rate has however not been reported in general.

Bevacizumab treatment has previously been reported to be a risk factor for bowel perforation, and a retrospective study from 2015 suggested a higher risk of perforation with stent treatment in patients treated with this drug (51) [3A]. The most common problem with stent as palliation is migration. Stent migration has an incidence of up to 10.5% (52) [2A]. Migration can be related to treatment with palliative chemotherapy (as the response triggered by treatment can result in tumor shrinkage) or to stent type and diameter. Migration rates are expected to increase with longer survival due to more effective palliative chemotherapy. However, a study comparing palliative treatment with stoma vs. stent treatment suggested a greater likelihood of discharge to home with stent treatment (53) [2B]. In a randomized trial between stents and stomas, the hospital length of stay was shorter, and the quality of life was higher in stent-treated patients (54) [1B]. Stenting is therefore a recommended choice for the palliative treatment of stenosing colon cancer.

Patients with colorectal cancer and colon perforation are frequently severely septic. The initiation of medical treatment for hypotension, metabolic acidosis and infection is recommended as soon as possible, as the severity of sepsis has a major impact on patient mortality and morbidity (C).

Primary oncologic resection of the bowel is recommended as the first surgical choice. If the patient's physiological condition, comorbidities and tumor location put them at high risk of anastomotic leakage, primary resection and stoma placement are recommended. A double lumen stoma (loop or split stoma) is recommended because it increases the possibility of closure (B).

Colonic perforation, a complication of obstructive colorectal cancer, is associated with high morbidity and mortality (55) [4]. The incidence of perioperative mortality was reported to be between 5% and 19% in a retrospective US study (56) [2b]. Perforation can be categorized as perforation of the colon proximal to the obstructing tumor site due to distention or perforation of the tumor itself. In the case of perforation of the tumor itself, abscess formation and local peritonitis may occur. Furthermore, studies suggest that tumor perforation is an independent risk factor for the development of peritoneal carcinomatosis (57, 58) [3b-3a].

Perforation of the colon proximal to the tumor frequently results in fecal peritonitis and severe septic conditions, which require urgent surgical intervention to control contamination and septic shock (59) [3b]. Sepsis severity has a major impact on postoperative mortality in patients with colorectal cancer and colon perforation (60) [3b]. It is therefore important to treat patients’ hypotension, metabolic acidosis and systemic inflammatory response as soon as possible. In the UK, a targeted intervention with “sepsis packages” has been shown to significantly reduce mortality (61).

There are various surgical options for patients with tumor perforation. Oncologic resection is recommended. The choice between primary anastomosis or stoma should depend on the degree of contamination, the patient's physiological condition,sepsis, comorbidity [American Society of Anesthesiologists (ASA) score] and tumor location. There is generally a higher risk of anastomotic leakage in acute surgery (59, 62) [3a]. Stoma placement should therefore be chosen for patients who are at high risk of postoperative anastomotic leakage and is expected in a higher proportion of emergency patients. Risk factors for anastomotic leakage include age, male sex, an American Society of Anesthesiologists (ASA) score >3, smoking status, diabetes status and a serum ALB concentration <4 g/dl (63) [2c]. Double-lumen stoma (loop or split stoma) is preferred, as it makes later stoma reversal more likely (56, 58, 59) [3b]. The fact that stomas that develop during emergency surgery have a lower probability of being closed should be taken into account (56) [3b]. For perforation of the coecum in the presence of right-sided colon tumors, right-sided hemicolectomy with ileocolic anastomosis or ileostomy is recommended. In the case of cecal perforation and a tumor located in the left colon, subtotal colectomy is recommended. If the perforation was in the left colon and the tumor was in the same location, left-sided hemicolectomy with primary anastomosis was recommended. Alternatively, Hartman's operation can be performed (58, 59) [3b].
